# Medical News and Miscellany

**Published:** 1895-10

**Authors:** 


### Medical News and Miscellany.

---

A labor-ious practice:—Obstetrics.

---

A great hardship:—An iron-clad; the Oregon, for instance.

---

Dr. F. S. White has removed from Houston to Fort Worth.

---

Dr. J. Angus Gillis has located at Buda, Hays county, Texas.

---

Dr. Joseph Meyers has removed from Honey Grove to Dallas.

---

Dr. A. J. Gray has removed from Ben Wheeler to Will's Point, Texas.

---

Gelseminum controls pain in the ovaries better than anything else, except narcotics.

---

The Committee on Banquet was composed of Drs. Baker,

Cook, Boyles, Broiles, Fry and Stew-ard. Dr. Sourwine was added by special request.

---

Quinine pills made with aromatic sulphuric acid can be broken up at time of using and readily given to children with a little brown sugar or chocolate.

---

**Foolhardy.**—Dr. Ira H. Hardy, of Tampa, attempted to operate for phymosis without an anæsthetic. The boy jerked, and the doctor sliced off a piece of the glans penis.

---

We are requested by Secretary West of the Texas State Medical Association to say that the Transactions will not be ready for delivery until some time in October, owing to delay in getting an important paper.

---

Physicians who contemplate taking a post-graduate course this fall, can learn something to their advantage by dropping a card to the managing editor of the JOURNAL, stating about when they expect to go.

---

Dr. Edmonson, of Dallas, makes a specialty of orthopedic surgery, and has established a hospital in that city for the treatment of all kinds of deformities. This is the first departure of the kind in the South, so far as we know, and we wish the doctor success.

---

**A Serious Scrape.**—At a village in Georgia, Dr. A. Q. Rhett, with an instrument of a similar name, scraped out the uterine cavity of a patient, and in a short time she aborted. (Didn't know it was loaded). Dr. Bledso had to be called in to assist in arresting the hemorrhage, she bled so.

---

**Memphis Medical Monthly.**—A monthly Journal of Medicine and Surgery. Established 1880. Supscription price $1.00 a year. Commencing with the November issue, will publish, each month, liberally illustrated, one or two of the best *clinical* lectures delivered in the Memphis Hospital Medical College. Write for sample copy.

---

The seventh annual meeting of the Tri-State Medical Society of Alabama, Georgia and Tennessee, will be held in Chattanooga, Tuesday, Wednesday and Thursday, October 8th, 9th and 10th. A large attendance is expected. An attractive programme will be annonced shortly. Papers have been promised by Drs. J. B. Murfree, J. E Woodbridge, J. B. Cowan, R. P. Johnson, W. E. Davis, J. C. LeGrand, Richard Douglas and R. M. Cunningham.

---

**And alas, Another.**—Dr. W. P. Burts, of Fort Worth, died in that city September 5th, ult., aged 69 years. Dr. Burts was another of the land marks of Texas medicine now rapidly passing away. He was President of the Texas State Medical Association in 1890–'91, and was at the time of his death Professor of Obstetrics in the Fort Worth Medical College. We hope to give an extended notice of his life and works, and a portrait in our next issue. His loss will be long and severely felt.

---

**Sterilize the Testicle.**—Dr. McCully, of Toronto, Canada, calls attention to a new treatment for prostatitis. He says (*Medical Record* Aug 24): "The one word sterilization of the testicle is in my experience the embodiment of success; the moment semen ceases to be developed and spermatozoa no longer manifest themselves, recovery commences. He uses an injection of cocaine into the body of the testicle, completely sterilizes the testicle. He thinks cold water would answer the same purpose.

---

**Southwest Texas Insane Asylum, San Antonio.**—Dr. T. C. Karnes has resigned the position of assistant physician at the Southwest Texas Insane Asylum, in consequence of paralysis of his arm, the result of a gun-shot wound mentioned in these pages a while ago. Dr. T. T. Jackson, late of Iredell, has been appointed to the position. Dr. Jackson is a graduate of the Texas Medical College (Medical Department University of Texas) of the class of 1891–'92, and took first honors, we believe. He has had an experience of three years as resident physician in the Sealy Hospital, which admirably fits him for the position he now occupies.

---

**The term** "Uricacidemia" recently was stigmatized in these columns as a fearful mongrel never employed by educated writers. This assertion, however, is not altogether true, it seems. For in a late number of the *British Medical Journal* the eminent Dr. Haig employs, unreservedly, this wonderful combination of two

English words and a Greek suffix. Hereafter no one will be surprised to see crop up in medical literature such vocabulary beauties as whitebloodcorpuscleemia, grapesugaruria, eggwhiteuria, gall-stoniasis, stomachguttitis, etc. Verily, the English language has lost nothing of the flexibility and adaptability of vigorous youth.—*Western Drurgist*

---

**Shortly after** you have finished reading this excellent number of the Red-back, doctor, and while you are still feeling good, realizing the benefit you have been deriving from its monthly visits all this long time, you may expect a little reminder that it takes something more than good wishes and kind words to get up, regularly every month, and mail out sharp on time, a bang up journal like this, sixty odd pages of live, spirited and very interesting reading, and forty odd of the best and handsomest advertisements extant; yes, sir, we have to plank down a big lot of money on the first of every month, and we are just obliged to look to our subscribers for an occasional remittance. We will mail out every bill that is due. Now, don't get mad, but send us a remittance, to make *us* feel good a little bit.

---

**Hon. Clark Bell.**—At the recent session of the International Medico-Legal Congress, held in New York, Ex-Surrogate Rastus S. Ransom presided and delivered an address. He paid a high tribute to the genius of Clark Bell, Esq., the President elect of the Society, for the interest he has always taken in maintaining its prestige, and for giving it a name and a fame in this and other lands. The above is a merited compliment; but we think the judge might have gone further and said that Clark Bell was the father of the Society; that he, almost unaided at first, had built it up from a handfull of members and a very local existence, to an International Society, the influence of which is felt and acknowledged throughout the civilized world. Its work will live forever, and in time will revolutionize our antiquated medical jurisprudence. All honor to Judge Bell.

---

**The Red Back** is not only red, but very extensively read. A subscriber who a short while ago contributed an article to its pages, writes us that he had to date, received one hundred and four letters on the subject, and they were still coming. He says, "I had no idea the JOURNAL was so extensively circulated. I have received letters from distinguished physicians in all parts of the country, from Maine to Mexico. I regret that I have not been a reader of the JOURNAL from the first.''

Now, this is a straw that correctly indicates the way the wind is blowing. The writer referred to is a prominent well known physician and a man of integrity and veracity. We withhold names, but the letter is on file in our office, and can be seen by any who are inclined to think we are exaggerating.

A JOURNAL so widely circulated and influential is the one for advertisers to put their money on; and what everybody reads everybody else ought to read. Send along your subscriptions, you new fellows.

---

**The Cold Bath in Typhoid Fever.**—Dr. William Osler, of Baltimore, read a paper on ''Five Years' Experience with the Cold Bath Treatment of Typhoid Fever.'' He quoted statistics, showing the death-rate to be 6.3 per cent under this form of treatment, which was considerably less than that obtained from any other method of treatment. Statistics from other hospitals throughout the world agreed with his. The Brandt system, he confessed, he had not followed fully: Markèdly asthenic cases; patients with very high temperature, rapid pulse, meteorism; cases in which severe complications, such as hemorrhage, perforation, pneumonia were present; cases in which the temperature remained under 102½° F.; and mild cases—all of these did not receive the cold bath. In these—fifty-eight cases—the death-rate was 10.2. The effects of the cold bath were not only antipyretic, but beneficial to the system in many ways. The dietary consisted wholly of milk, broths, and egg albumin. The essayist then referred to particular cases, pointing out any special features in the way of symptoms or complications. He outlined, in reply to question, the way in which the baths were given. Many cases of typhoid, he believed, died from over-medication—from too active antipyretic treatment, from too much digitalis, or too much nitro-glycerine, or too many doctors.—*Medical Record.*

---

**Imperforate Hymen.**—Dr. D. L. Peeples, of Navasota, writes to the JOURNAL of a case of retained menses in a girl of fourteen who experienced her first menstrual molimen about April 30, 1895. There was no ''show'' at the time, but there was, he says, sufficient suffering to demand the attention of a physician. One was called, but he failed to correctly diagnose the case, and failed to give relief. At the second period the symptoms were intensified, and another doctor was called, who diagnosed the case "renal colic." The girl continued to suffer during the interval of the second and third periods, and she became greatly emaciated. On June 30th Dr. Peeples was called to the case in great haste. He found severe headache, lumbago, abdominal pains, vesical and rectal tenesmus, fever, muscular tremors, flashes of heat, vertigo, and extreme tenderness over the abdomen, which was greatly enlarged. She was threatened with convulsions. Upon examination he found great abdominal distension, and though suspecting retained menses, palpation led him, he says, "to suspect ovarian fibroma of left ovary, pyo-salpingitis of the left fallopian tube; the left tube and womb could be distinctly outlined." Upon digital examination he discovered great hyperæsthesia and a distended and imperforate hymen. The hymen was remarkably thick and hyperplastic. The vaginal examination was made under an anæsthetic, of course. Dr. Peeples made an incision through the hymen, and a large quantity—perhaps a quart—of dark, thick and tarry semi-fluid was discharged. All the symptoms were at once relieved. The parts were then washed out with a bichloride solution, 1 to 2000, and the canal dusted with iodoform. No packing was used, and the patient was discharged with a normal flow and rapidly recovered.

---

**Eczema.**—Here is a case of a little child of one year with an eruption that is papular, each papule sitting on a reddened base, the eruption scattered over the whole body. On the summit of each papule there appears either an excoriation or a crust. The child scratches and its nails, embedding themselves into its skin, open the hyperæmic papillæ, a minute hemorrhage occurs and the dark little scabs are formed. There is a little crusting on the sides of the abdomen, none on the face and much on the head. The fact that there is so much on the head leads to a diagnosis. Eczema is a protein disease, and shows itself in different stages of development on the affected skin. We notice that the hairs are matted together, and greenish crusts—the result of dried up pustules—have formed. This is the prototype of eczema. It is eczema papulosum of the body and eczema pustulosum of the scalp. With eczema papulosum there is more itching than with other eczemas. It is most obstinate. The itching is caused by the hard papule pressing on the sensory nerve filaments. This eruption is paroxysmal. She has had it more or less for seven months. Last night it re-occurred in an active form, causing great itching and restlessness. It often depends on faulty alimentation, but not in this case, as the child is breast-fed. But in strumous children and in adverse sanitary conditions we are apt to find this form of eczema. There are papules on the tongue of this child resembling thrush of a papular kind. It corresponds to the eruption on the skin. If the child gets nothing but mother's milk, I should advise the following:

*Treatment.*--An alkaline bran bath, or an oatmeal bath. The oatmeal bath is convenient. Place the raw cracked oatmeal in a well-washed salt sack; tie and press it out in the water until the water turns milky white and has a mild, oily touch; then add two teaspoonfuls of household ammonia. Give a bath once a day, before the child goes to sleep. Rinse off, dry and apply the following mild lotion:

℞ Zinci oxidi,

Calamine............aa ʒii

Glycerini............ ʒi

Aquæ calcis,

Aquæ...............aa ℥iii

Take a piece of cotton and daub the whole child with this lotion and allow it to dry. The child's bowels are normal, and it takes mother's milk, so that is all that is necessary. After the itching is allayed, Lassar's paste may be used five or six days from now:

℞ Acidi salicyl ...............ʒss

Pulv. zinci oxidi,

Pulv. amyli ............aa ʒvi

Vaseline................ ℥i ss

It is a useful remedy, and will do in the second stage of this disease. Internally, I recommend 1-10 of a grain of a calomel triturate twice a day and nursing at regular intervals.—*Dr. L. Weiss, in Post-Graduate.*

---

### A Few Selected Prescriptions.

---

CHRONIC RHEUMATIC ARTHRITIS.—Lord Anson paid £300 for the privilege of publishing the following prescription for chronic rheumatic arthritis:

℞. Sulphur..............................℥j.

Cream of tartar.....................℥j.

Rhubarb..............................℥iv.

Gum guiac..........................℥j.

Make one powder and add honey.......℥xvj.

Mix well, take two tablespoonfuls in a tumbler of white wine and hot water on going to bed, and repeating the dose on getting up in the morning.—*Louisville Medical Monthly.*

---

ACUTE CORYZA.—

℞. Cocain. muriat........................gr. vj.

Bismuth. subcarb.......................ʒ ss.

Talc...................................ʒ jss.

M. Sig: Enough to cover a silver five-cent piece insufflated into each nostril every two hours.—SAJOUS.

---

℞ Carbolic acid.........................pt. j.
Ammonia water..........................pt. j.
Alcohol................................pt. ij.
Distilled water........................pt. iij.

Mix. Sig.: Gtt. 10 to be dropped on a piece of blotting-paper and inhaled for a few moments every hour.—*Monit. Therap.*

---

NASAL AND FAUCIAL CATARRH.—

℞. Acid. carbol. liq....................*M* xxx.

Sodii biborat,

Sodii bicarb...........................aa ʒ j.

Glycerinæ..............................f ℥ iijss.

Aquæ...................................q. s. ad f ℥ iv.

M. Sig.: To be used as a spray.—DOBELL.

---

CHRONIC DIARRHŒA AND DYSENTERY.—

℞ Cupri sulphat.,
Morphiæ sulphat........................aa gr. i.
Quiniæ sulphat.........................gr. xxiv.

M. ft. pil. No. XII.

Sig. One pill three times a day.

---

SQUIBB'S CHOLERA MIXTURE.—

℞ Tinct. opii,

Tinct. capsici,

Spts. camphoræ.........................aa fl. ʒi.

Chloroformi............................fl. ʒiii.

Alcoholis..............................q. s. ad fl. ʒv.

M. Dose.—Twenty to forty minims.

FLATULENT COLIC.—

℞ Tinct. nucis vomicæ ............... ʒi.

Acidi nitro muriatici dil............ ʒii.

Spiritus chloroformi................ ʒi.

Infus. gentianæ...................ad ℥vi.

Dose.—Tablespoonful three times daily after meals.

---

ACNE ROSACEA.—

℞ Sulphur præcip..................... ʒj.

Calaminæ præpar.................. ʒij.

Zinci oxidi.......................... ʒj.

Glycerini ........................... ʒj.

Aquæ destil.....................ad ʒiv.

M. ft. lotio.

Sig. The lotion to be shaken, then painted on with a camel's hair brush at night.

In morning face is washed with a little warm water (no soap) and powdered over with following:

℞ Acidi borici..................... pts. x.

Talci ........................... pts. xv.

M. ft. pulv.

Sig. To be applied every morning.—JAMIESON.

---

### The Treatment of Gonorrhea in Women.

Dr. William R. Pryor read the paper on this subject. He said that throughout the entire treatment of gonorrhea in women one principle must govern the physician; the disease must be locally checked and extension prevented. Gonorrhea produced few destructive lesions when it affected primarily the external genitals, but when it had extended to the pelvic viscera it often destroyed the integrity of the affected organs and chronic invalidism might be induced.

*Gonorrheal Urethritis.*—This should be treated from the very first. Internally he administered citrate of potash for the first three days or so, to alkalinize the urine, locally he applied every day nitrate of silver, 10 grains to 1 ounce, and after the urethritis had somewhat subsided the solution might be reduced to 5 grains to 1 ounce, and applied less often. The patient should frequently bathe the vulva with one-half per cent lysol-solution; but no douching was admissible.

*Gonorrheal Cystitis.*—Forty to sixty grains of benzoate of soda should be given internally, the bladder should be washed out with super-saturated solution of boracic acid at least twice a day, the bladder being filled and emptied several times at each sitting. The viscus should then be left full of the solution. The accompanying urethritis should be treated with daily application of nitrate of silver, 5 grains to 1 ounce. In washing out the bladder a single catheter should be used.—*Medical Record.*

